# Supplementary material for: Guaianolide Derivatives from the Invasive Xanthium spinosum L.: Evaluation of Their Allelopathic Potential
Source: Molecules. 2022 Oct 27;27(21):7297. doi: 10.3390/molecules27217297 (PMC9656820; doi:10.3390/molecules27217297)
Supplement: Supplementary file 1 [file molecules-27-07297-s001.zip › molecules-1919878-supplementary.pdf]

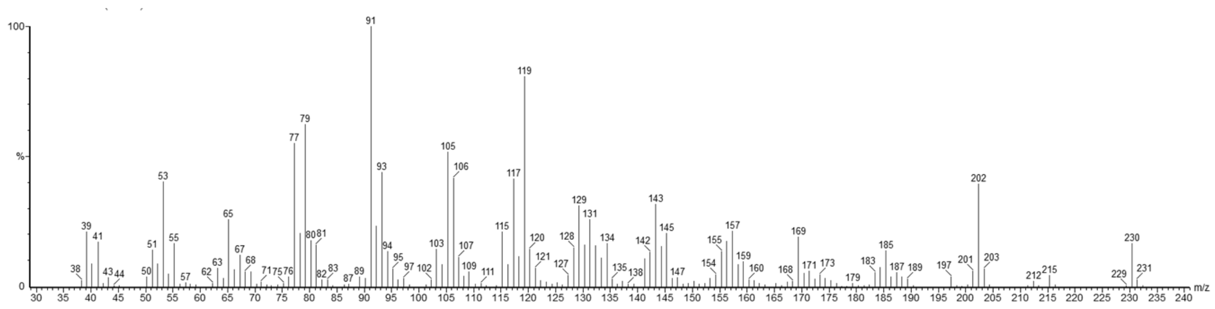


**Figure S1**. EI MS spectrum of **66**.

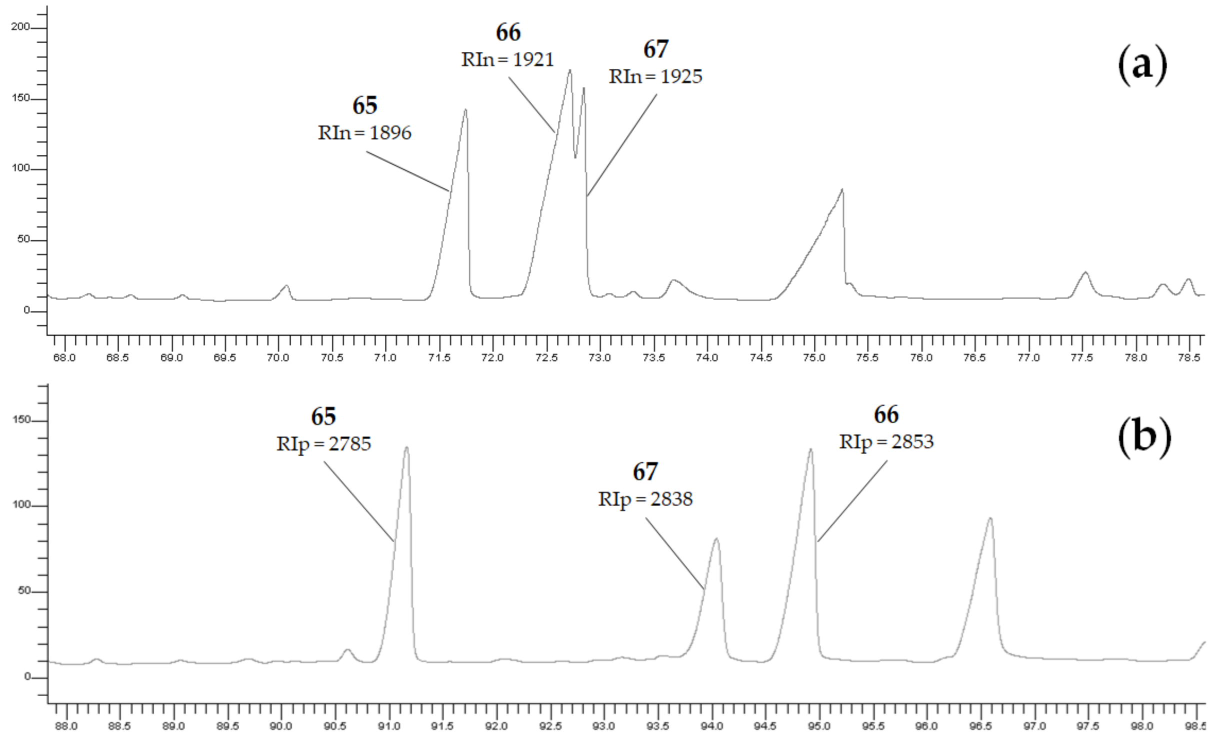


**Figure S2.** GC DIF Spectrum centered on compounds **65-67**. (**a**) non polar column. (**b**) polar column.

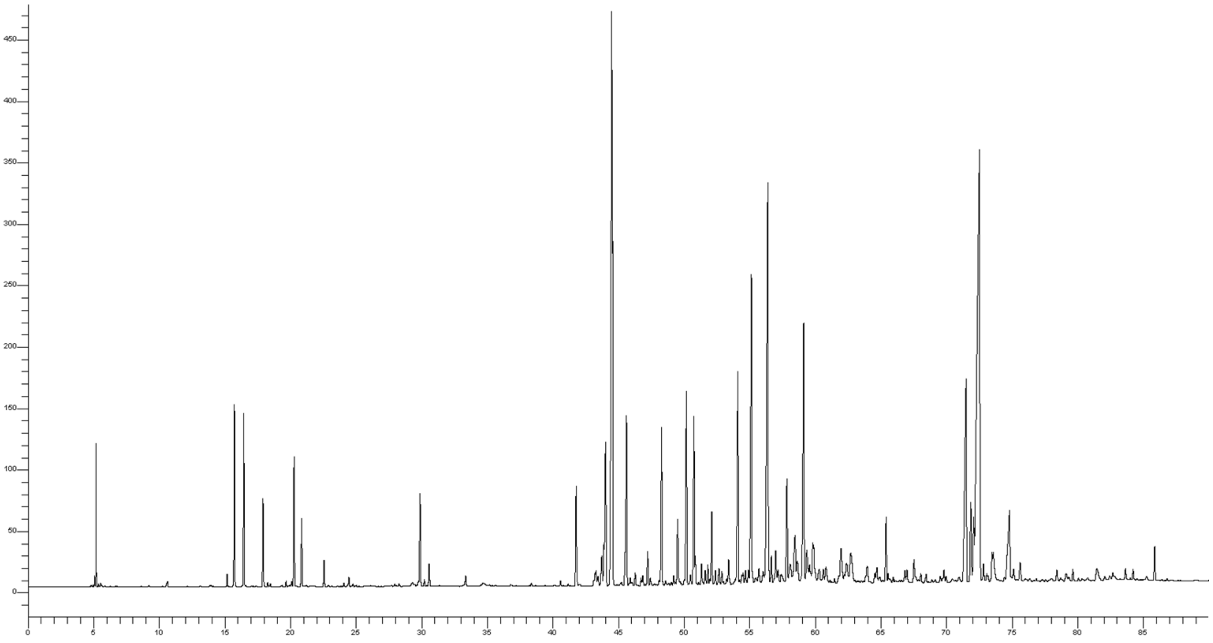

**Figure S3.** GC DIF Chromatogram of the EO.

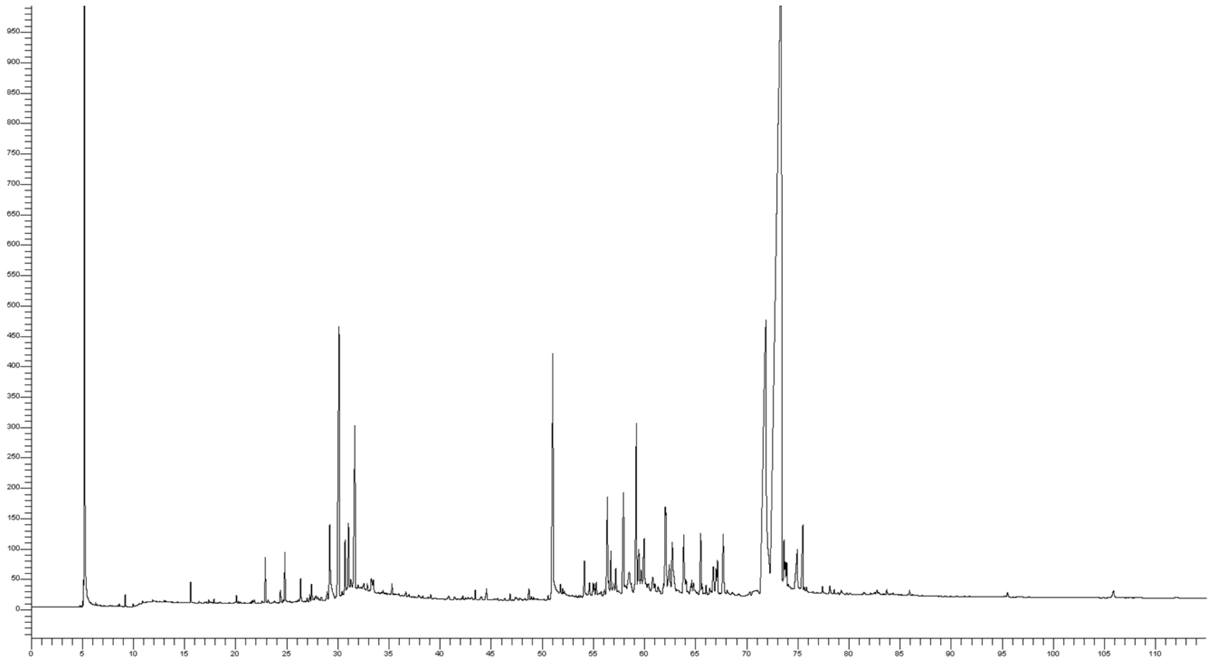

**Figure S4.** GC DIF Chromatogram of the HYD.

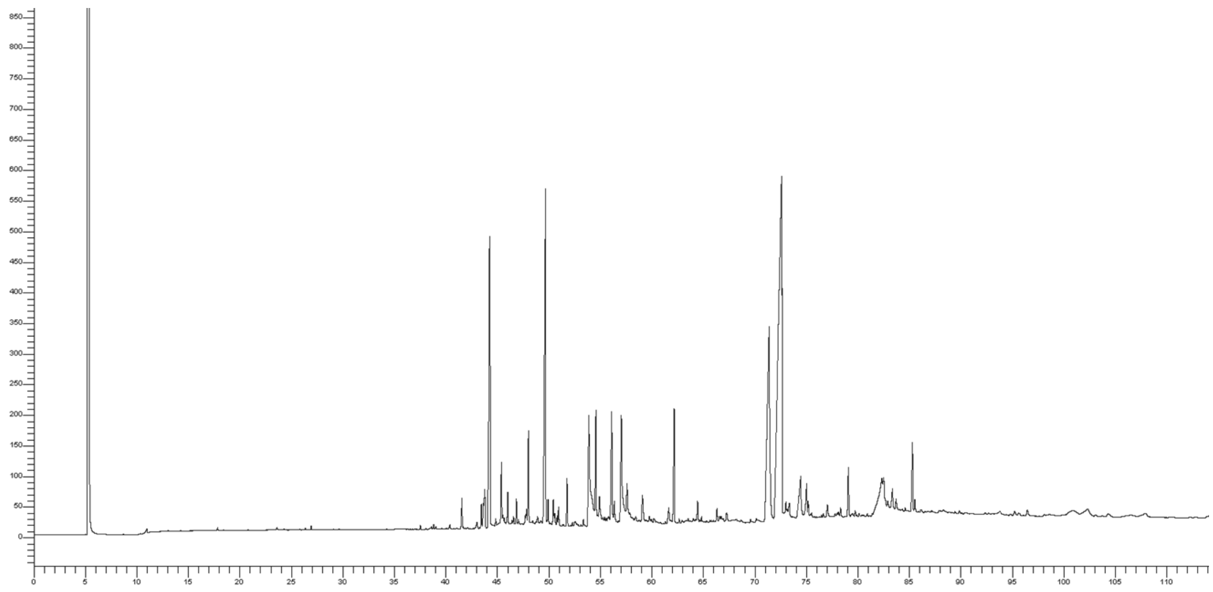


**Figure S5.** GC DIF Chromatogram of the MAC.

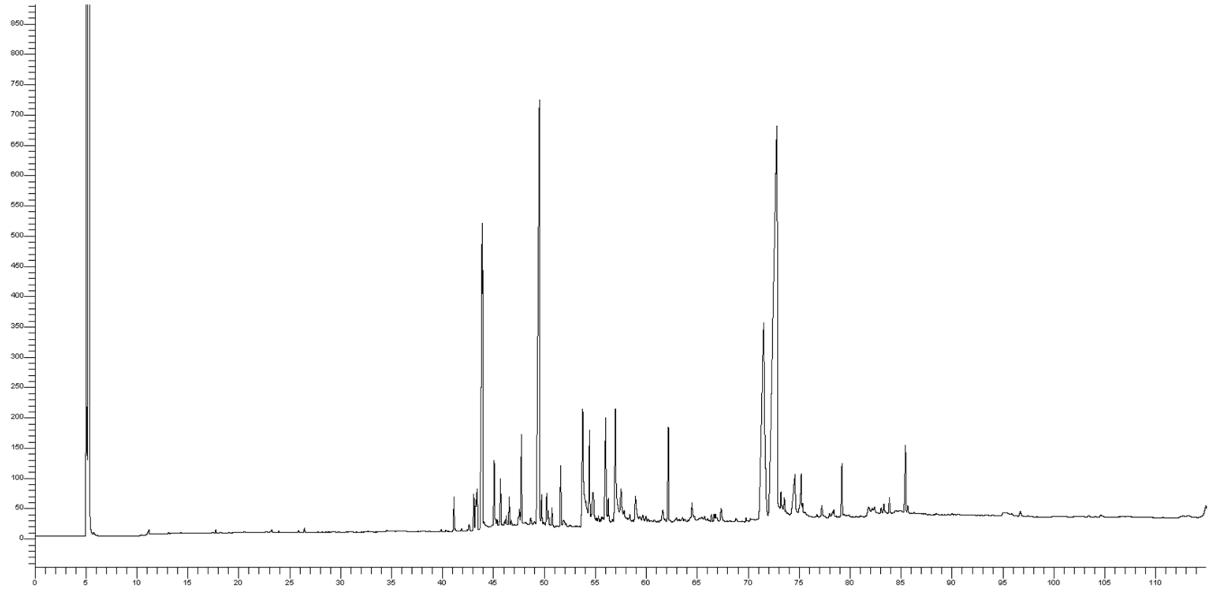


**Figure S6.** GC DIF Chromatogram of the MAE.

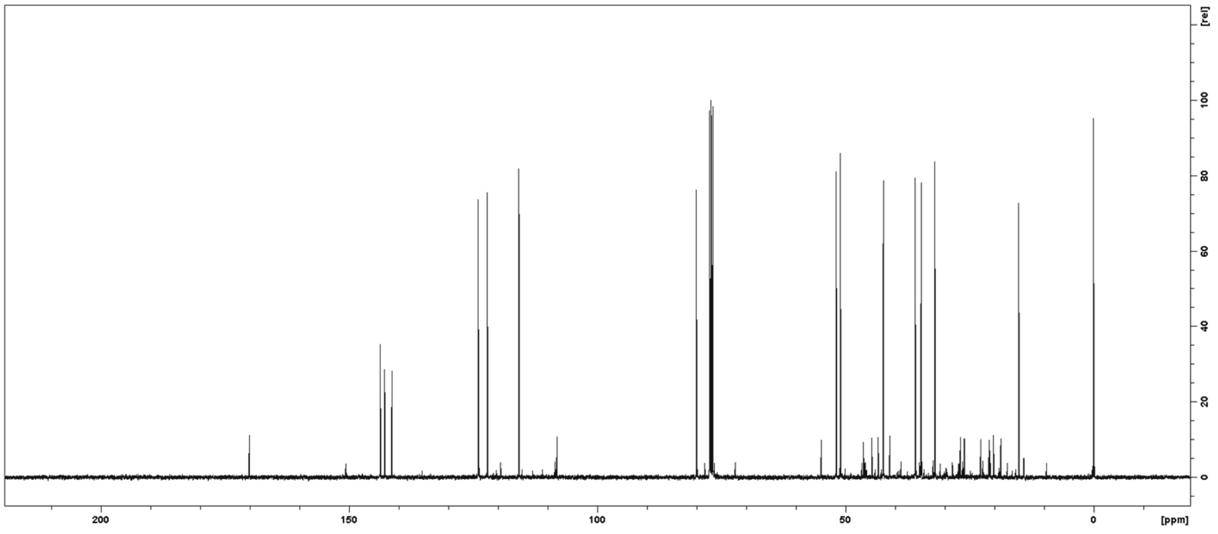


**Figure S7.** RMN $^{13}C$ Spectrum of **66.**

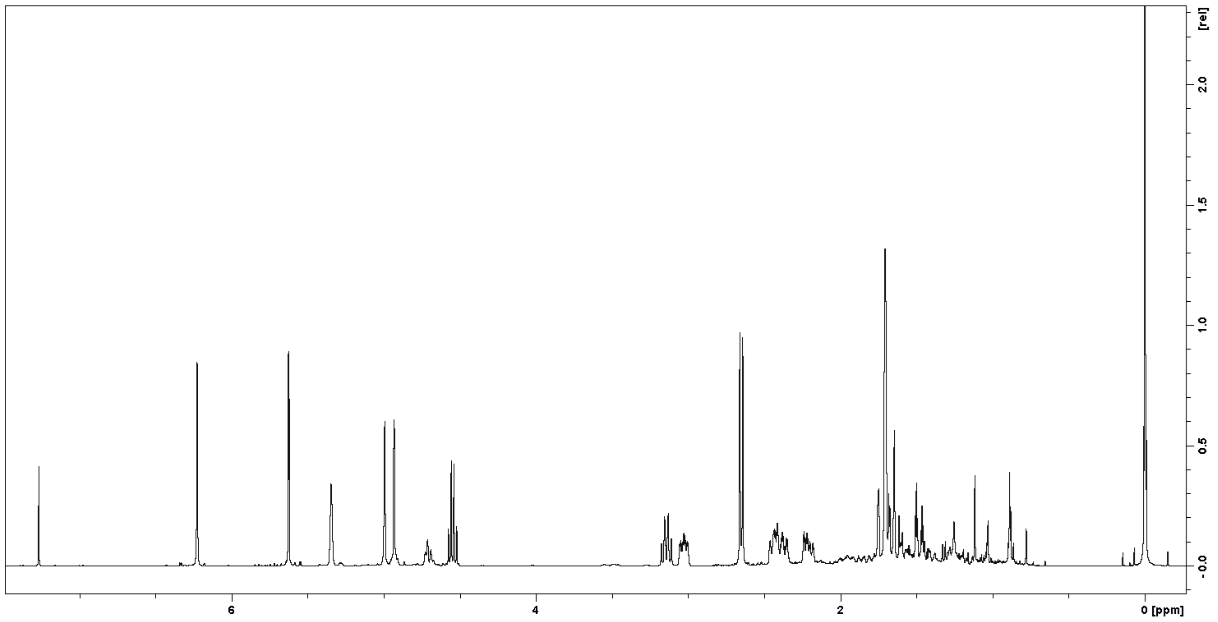


**Figure S8.** RMN $^{1}H$ Spectrum of **66.**

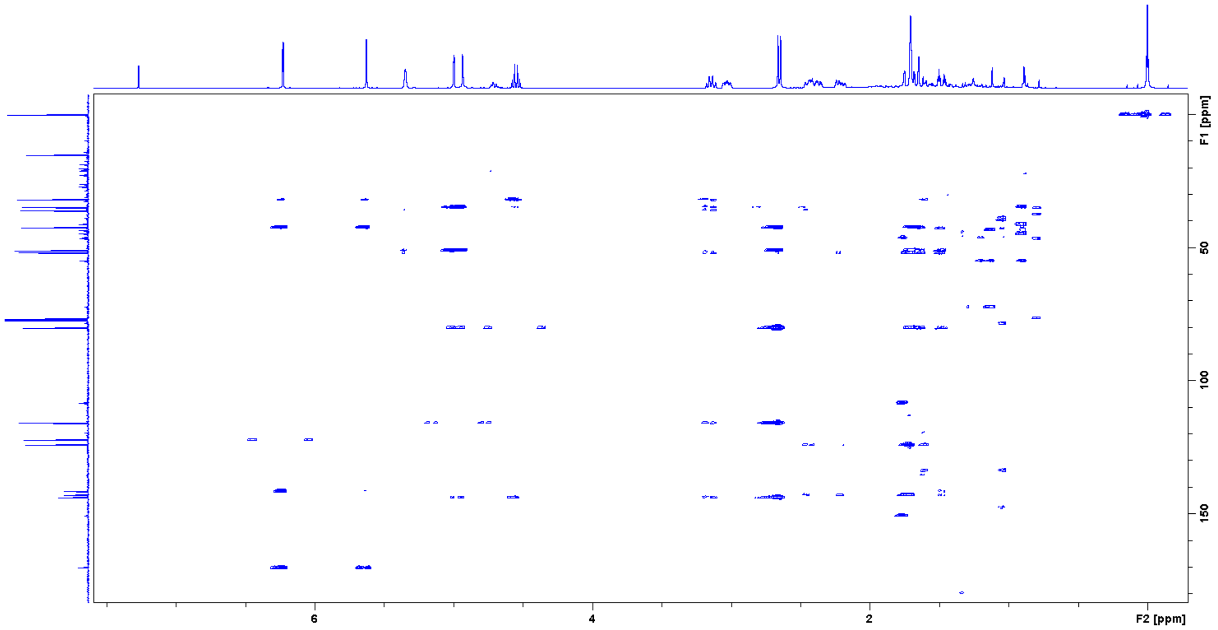


**Figure S9.** RMN HMBC Spectrum of **66.**

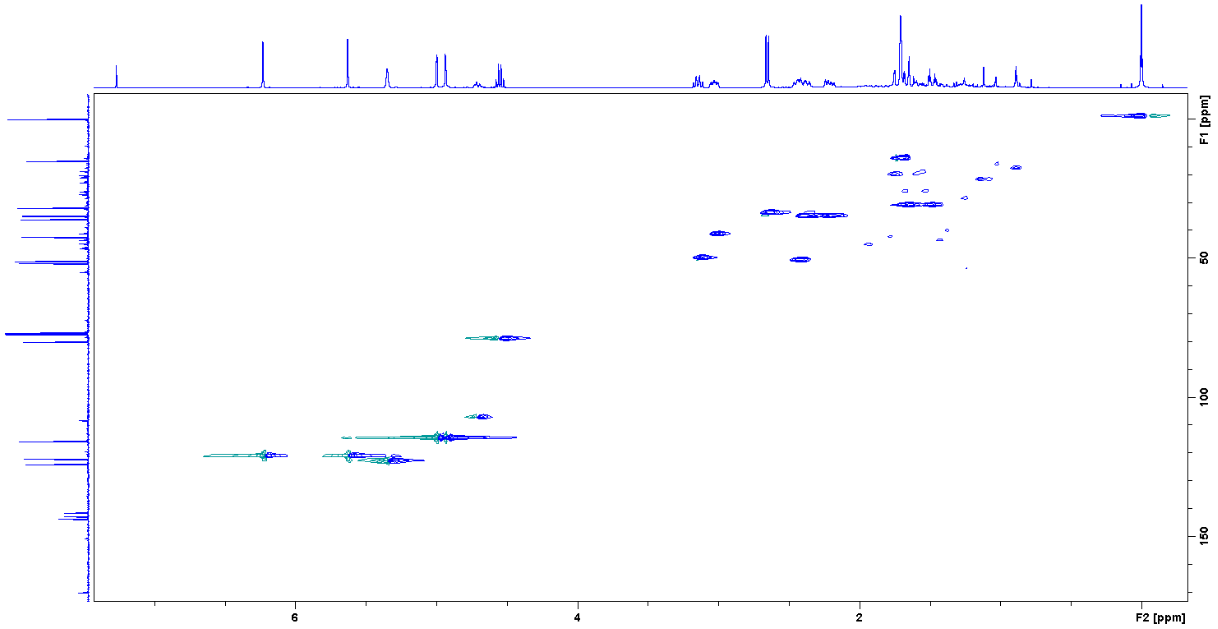


**Figure S10.** RMN HSQC Spectrum of **66.**

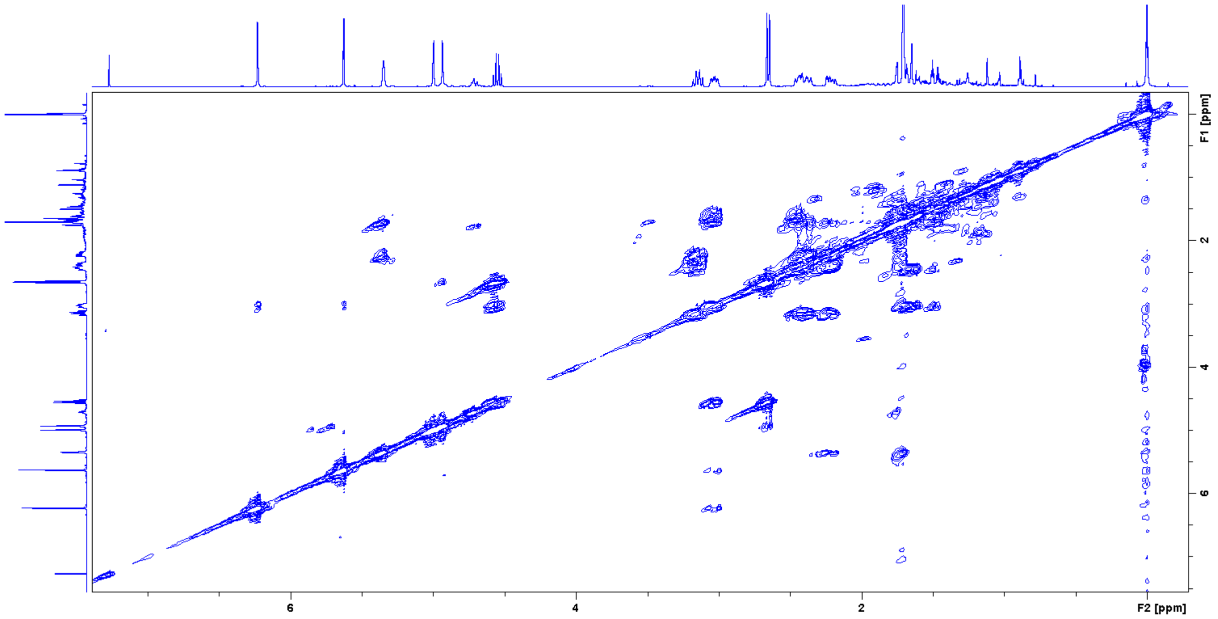


**Figure S11.** RMN COSY Spectrum of **66.**

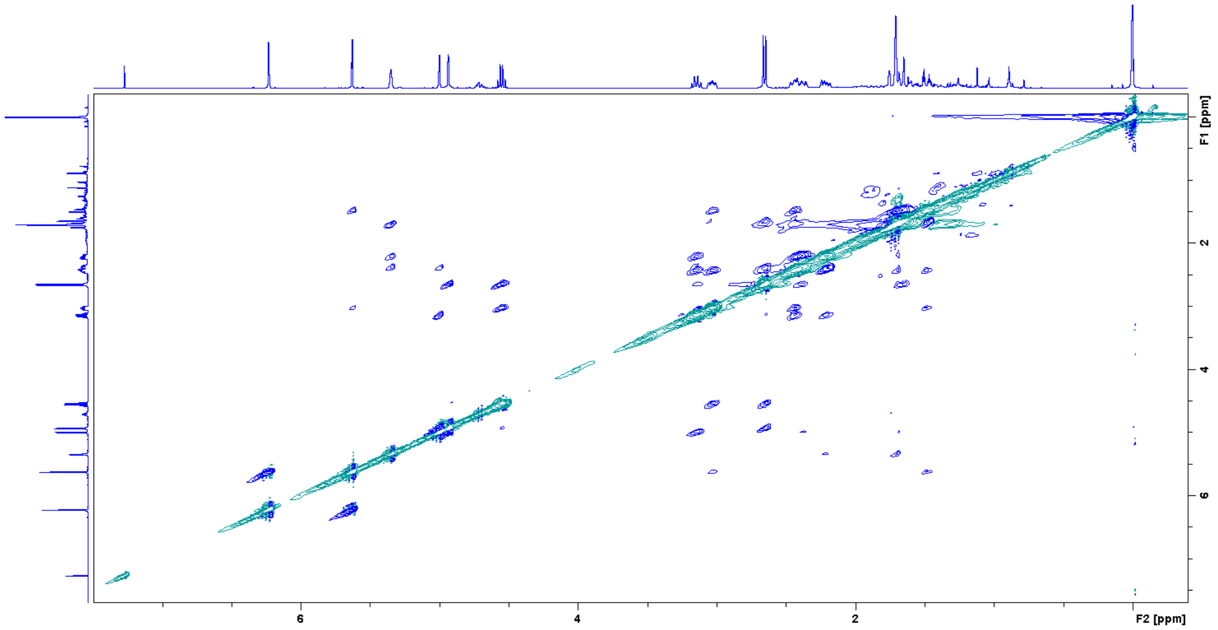


**Figure S12.** RMN NOESY Spectrum of **66.**

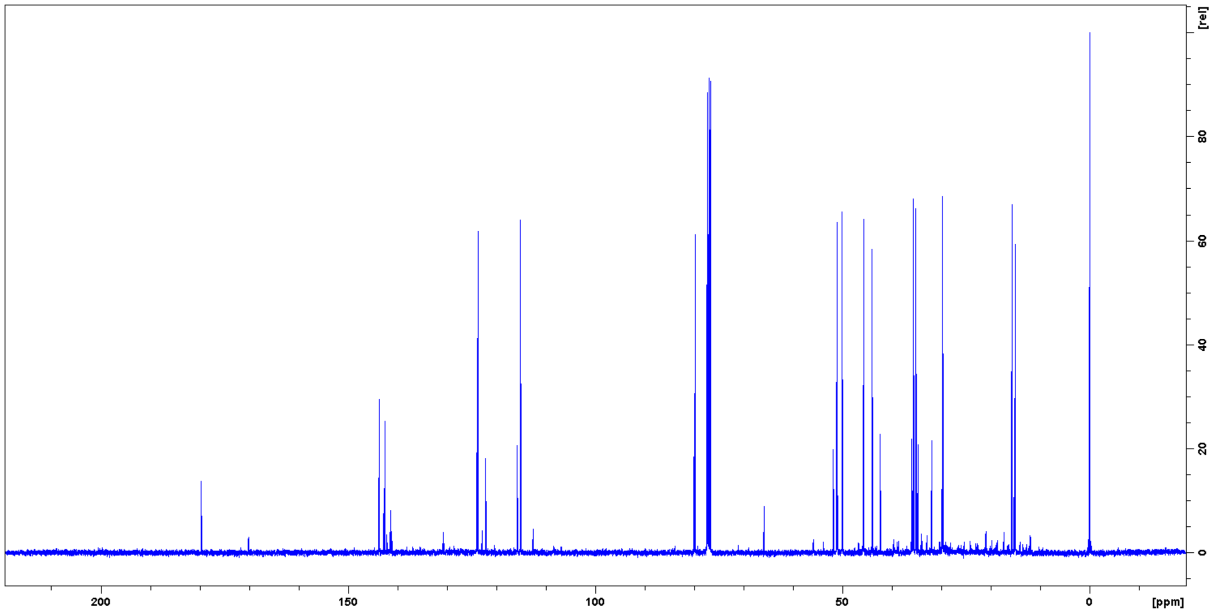


**Figure S13.** RMN $^{13}C$ Spectrum of **65.**

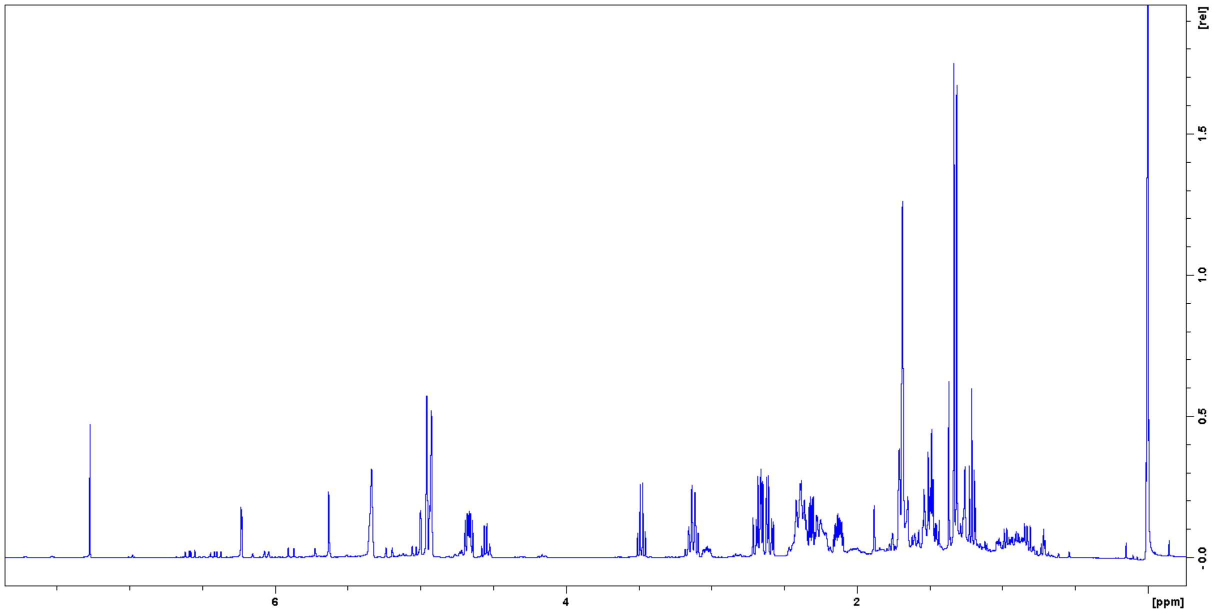


**Figure S14.** RMN $^{1}H$ Spectrum of **65.**

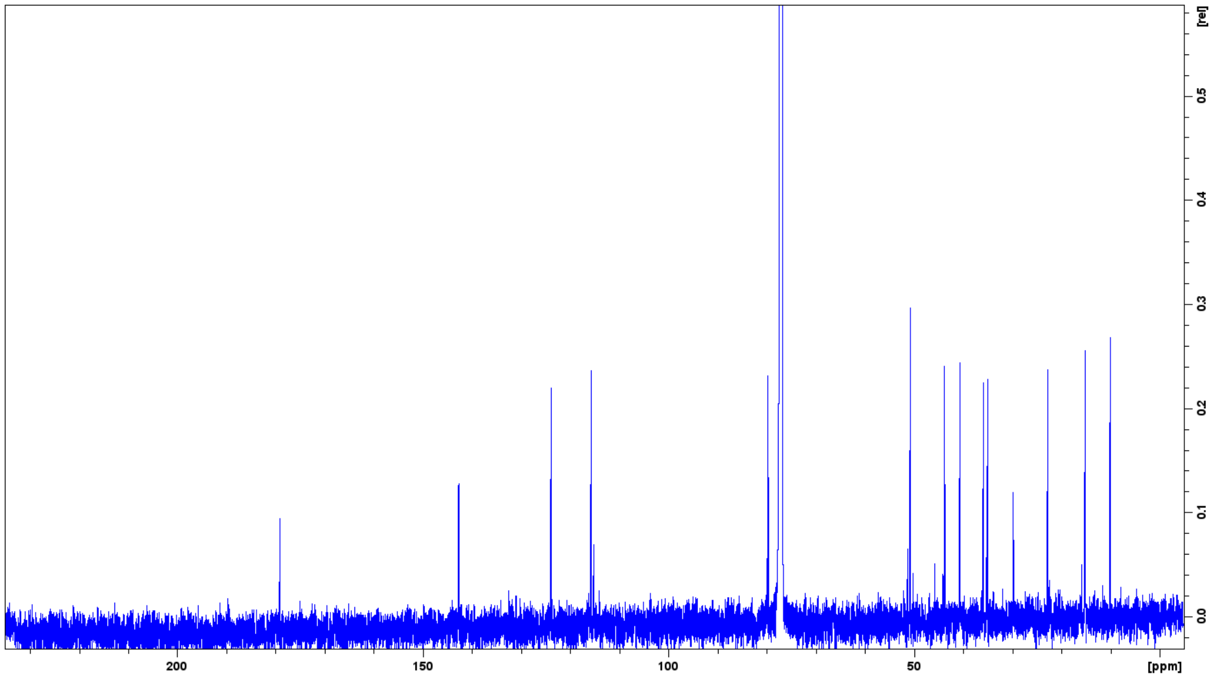


**Figure S15.** RMN $^{13}C$ Spectrum of **67.**

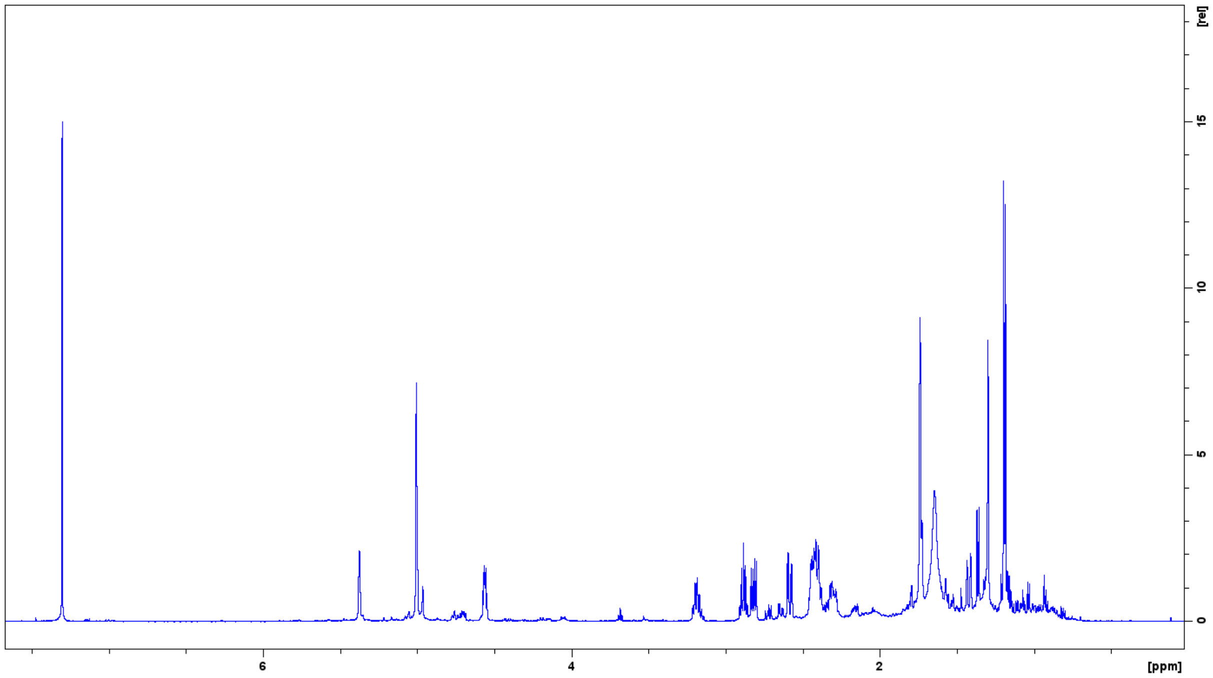


**Figure S16.** RMN $^{1}H$ Spectrum of **67.**

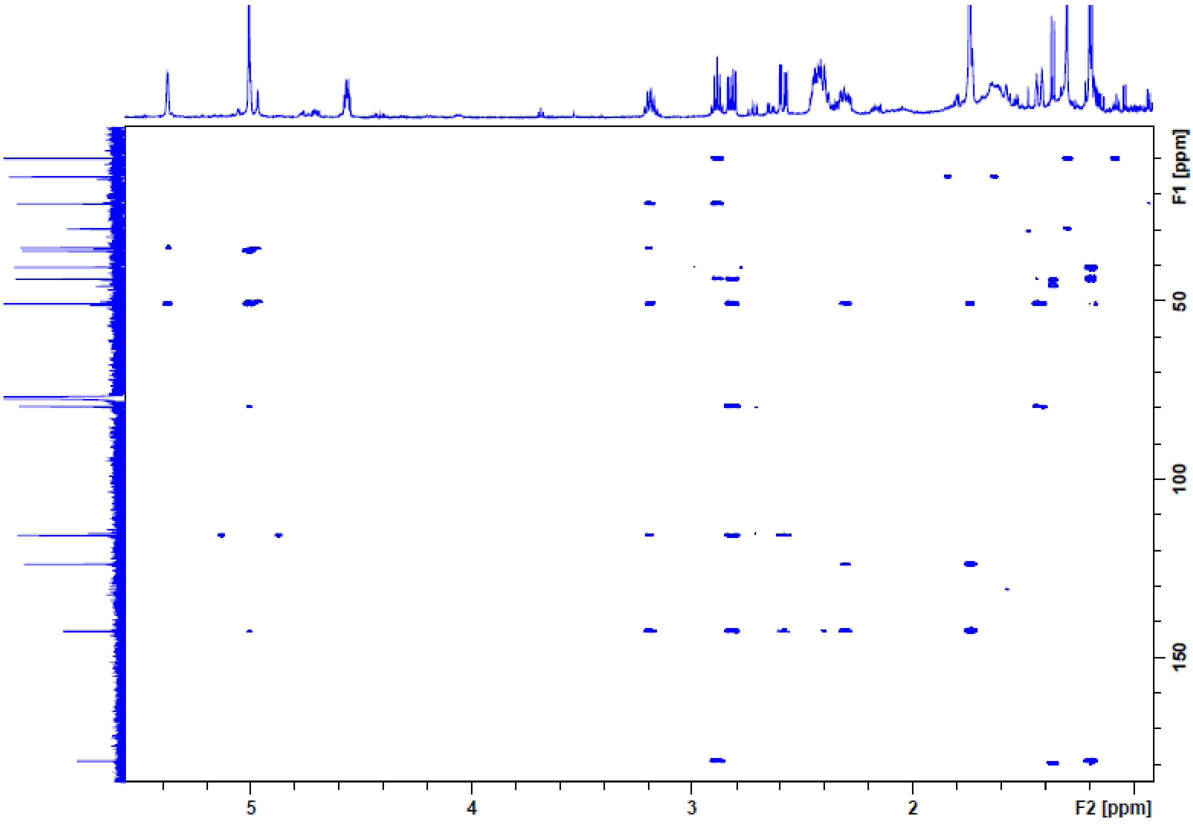


**Figure 17.** RMN HMBC Spectrum of **67.**

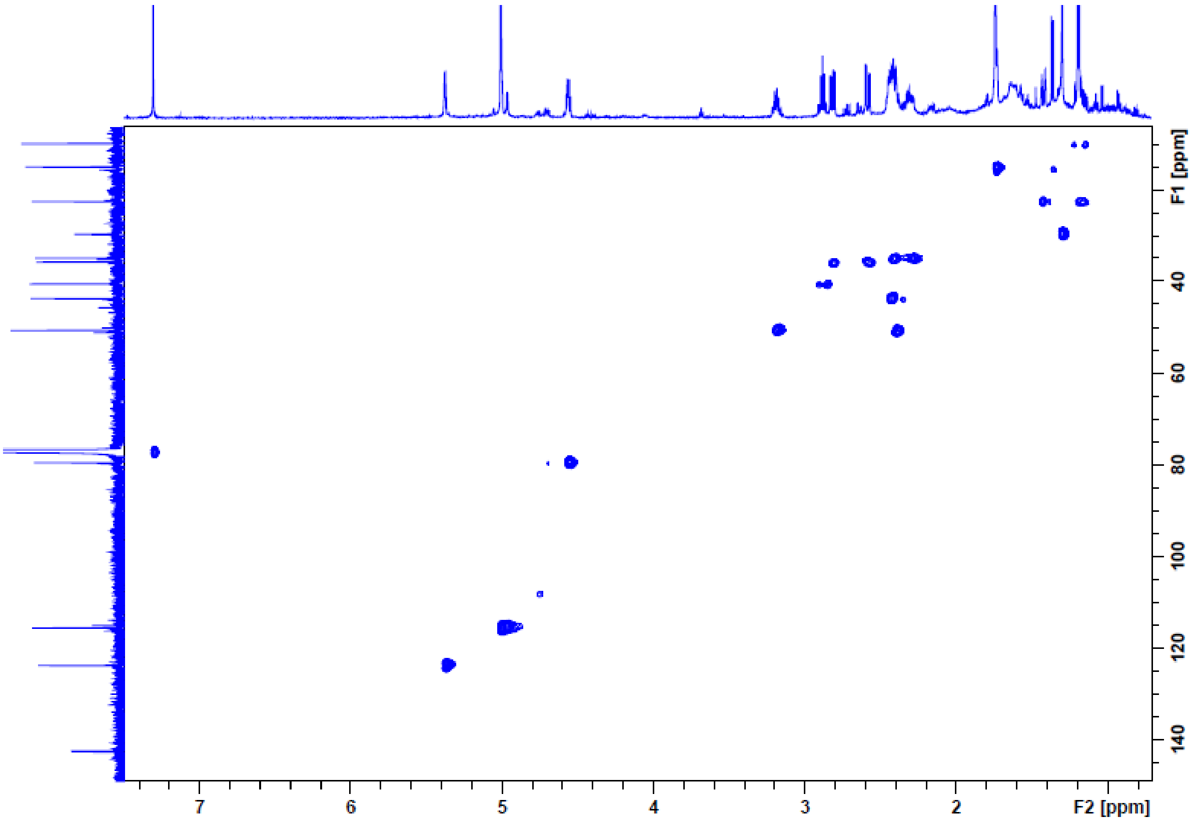


**Figure S18.** RMN HSQC Spectrum of **67.**

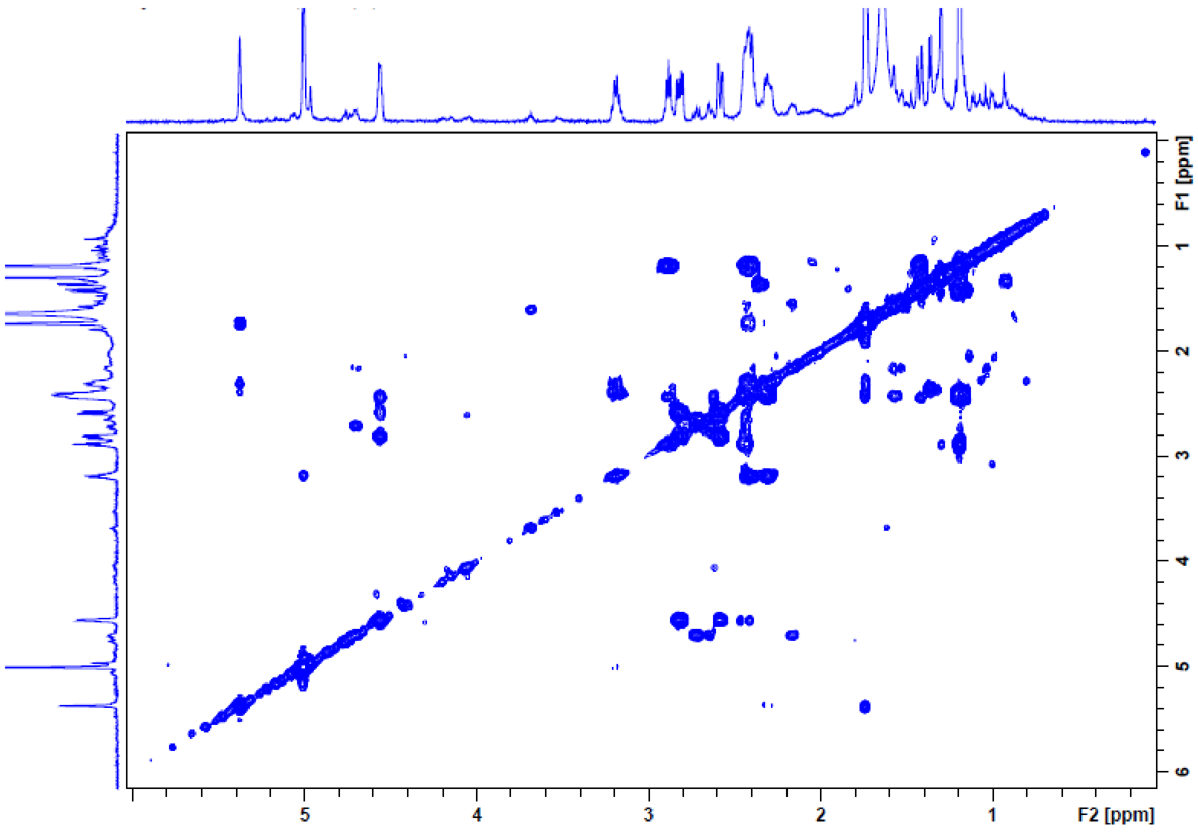


**Figure S19.** RMN COSY Spectrum of **67.**

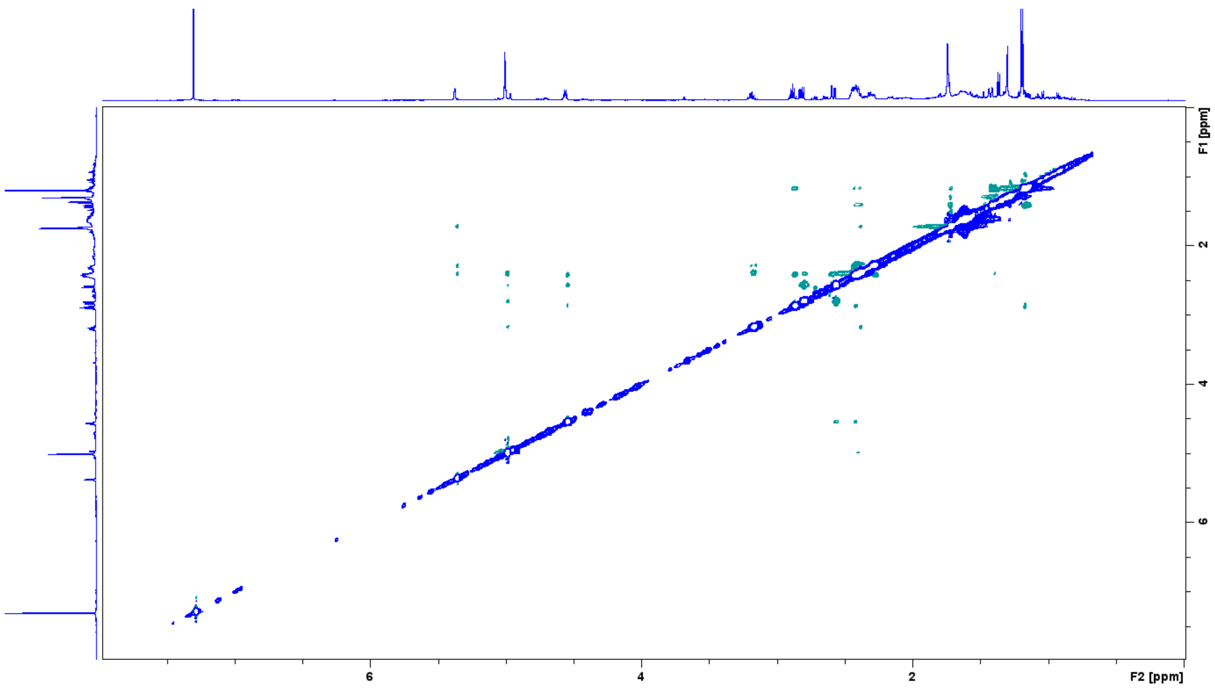


**Figure S20.** RMN NOESY Spectrum of **67**.
